# Development of a Recombinase Polymerase Amplification Assay for Schistosomiasis Japonica Diagnosis in the Experimental Mice and Domestic Goats

**DOI:** 10.3389/fcimb.2021.791997

**Published:** 2021-11-16

**Authors:** Qinghong Guo, Kerou Zhou, Cheng Chen, Yongcheng Yue, Zheng Shang, Keke Zhou, Zhiqiang Fu, Jinming Liu, Jiaojiao Lin, Chenyang Xia, Wenqiang Tang, Xiaonan Cong, Xuejun Sun, Yang Hong

**Affiliations:** ^1^ National Reference Laboratory for Animal Schistosomiasis, Shanghai Veterinary Research Institute, Chinese Academy of Agricultural Sciences, Shanghai, China; ^2^ Key Laboratory of Animal Parasitology of Ministry of Agriculture, Shanghai Veterinary Research Institute, Chinese Academy of Agricultural Sciences, Shanghai, China; ^3^ Institute of Animal Science, Tibet Academy of Agricultural and Animal Husbandry Science, Lhasa, China; ^4^ Huancui Development Center for Animal Husbandry, Weihai, China

**Keywords:** schistosomiasis japonica, recombinase polymerase amplification (RPA), real-time PCR, diagnosis, domestic goats

## Abstract

Although the prevalence of schistosomiasis japonica has declined gradually in China, more accurate and sensitive diagnostic methods are urgently needed for the prevention and control of this disease. Molecular diagnostic methods are advantageous in terms of sensitivity and specificity, but they are time-consuming and require expensive instruments and skilled personnel, which limits their application in low-resource settings. In this study, an isothermal DNA amplification assay and recombinase polymerase amplification (RPA) combined with lateral flow dipstick (LFD) were set up. It was used to detect *S. japonicum* infections in experimental mice and domestic goats by amplifying a specific DNA fragment of *S. japonicum*. The lower limit of detection for the LFD-RPA assay was evaluated using dilutions of plasmid containing the target sequence. Cross-reactivity was evaluated using genomic DNA from eight other parasites. The effectiveness of the LFD-RPA assay was verified by assessing 36 positive plasma samples and 36 negative plasma samples from mice. The LFD-RPA assay and real-time PCR were also used to assess 48 schistosomiasis japonica-positive plasma samples and 53 negative plasma samples from goats. The LFD-RPA assay could detect 2.6 femtogram (fg) of *S. japonicum* target DNA (~39 fg genomic DNA of *S. japonicum*), only 10-fold less sensitive than real-time PCR assay. There was no cross-reactivity with DNA from the other eight parasites, such as *Haemonchus contortus* and *Spirometra*. The whole amplification process could be completed within 15 min at 39°C, and the results can be observed easily using the LFD. The sensitivity and specificity of the LFD-RPA assay were 97.22% (35/36, 95% CI, 85.47%–99.93%) and 100% (36/36, 95% CI, 90.26%–100%) in mice, and 93.75% (45/48, 95% CI, 82.80%–98.69%) and 100% (53/53, 95% CI, 93.28%–100%) in goats. By comparison, the sensitivity and specificity of real-time PCR were 100% (36/36, 95% CI, 90.26%–100%) and 100% (36/36, 95% CI, 90.26%–100%) for mice, and 97.92% (47/48, 95% CI, 88.93%–99.95%) and 100% (53/53, 95% CI, 93.28%–100%) for goats. The LFD-RPA assay exhibits high sensitivity and specificity for the diagnosis of schistosomiasis japonica, and it is an alternative method for diagnosis schistosomiasis japonica in low resource setting.

## Introduction

Schistosomiasis is an important parasitic disease caused by trematode flukes of the genus *Schistosoma*, which are mainly distributed in tropical and subtropical regions (Loverde, 2019). It is listed as one of the six major tropical diseases for key research, detection, and prevention (Boatin et al., 2012). Schistosomiasis has a major impact on human health and socioeconomic development. It affects nearly 240 million people worldwide, more than 700 million people live in endemic areas, and approximately 41,000 people die from *Schistosoma* infection each year (Zhou et al., 2005a; Satrija et al., 2015). The three major *Schistosoma* species cause human schistosomiasis, including *Schistosoma japonicum*, *S. mansoni*, and *S. haematobium*. *Schistosoma japonicum* is mainly distributed in China, the Philippines, and Indonesia (Loverde, 2019).

*Schistosoma japonicum* has more than 40 known hosts, all of which excrete faeces containing parasite eggs that contaminate water sources and cause disease transmission. In China, cattle and goats are main sources of transmission, and only five cases of human were positive by schistosomiasis pathogenic examination in 2019 (Chen et al., 2014; Zhang et al., 2020). Some measures taken by the government have resulted in great progress in controlling schistosomiasis japonica of animal in China (Utzinger et al., 2005; Hotez et al., 2014). The prevalence and infection intensity of *S. japonicum* of animal have declined gradually, but more sensitive and accurate diagnostic methods are urgently needed for the prevention and control of this disease (Wang et al., 2018).

Traditional diagnostic methods for schistosomiasis japonica mainly include parasitological and immunologic diagnoses. Parasitological methods are the “gold standard,” but they are time-consuming and have a high rate of false-negative results at low-intensity infections (Yu et al., 2007). Immunologic methods are prone to higher rates of cross-reactive and false-positive results, and they are difficult in distinguishing current infections and previous infections (Wu, 2002; Xu et al., 2011). In addition, imaging techniques could be used in schistosomiasis diagnosis, such as observing hepatic fibrosis by ultrasonography (Maezawa et al., 2018). Molecular diagnostic methods that can diagnose diseases accurately, possess higher sensitivity and specificity, and produce lower cross-reactivity with other pathogens are therefore needed. Many studies have indicated that certain specific nucleic acid sequences of *S. japonicum* can be detected in the blood and faeces of infected hosts (Lier et al., 2008; Gordon et al., 2015). Therefore, it is feasible to diagnose schistosomiasis japonica by detecting specific nucleic acid sequences.

Standard nucleic acid detection methods, such as general PCR and real-time PCR, require laboratory equipment and sufficiently skilled operators. The loop-mediated isothermal amplification (LAMP) method is convenient, but complicated primer design and aerosol contamination during amplification limit its application (Xu et al., 2010; Xu et al., 2015). Recombinase polymerase amplification (RPA) is an isothermal DNA amplification technology that can complete the amplification reaction within 20 min at temperature (25°C−45°C). Products of DNA amplification can be detected by a lateral flow dipstick (LFD), which makes the endpoint analysis more flexible and useable in low-resource settings (Crannell et al., 2014; Rostron et al., 2019; Hu et al., 2020; Lei et al., 2020). In previous studies, RPA combined with LFD (LFD-RPA) was found to save time and achieve high sensitivity, high specificity, and convenience, and visual detection of some diseases can be employed (Rosser et al., 2015; Poulton and Webster, 2018; Wang et al., 2019; Shelite et al., 2021). Sun et al. set up the LFD-RPA method targeting SjR2 of *S. japonicum* to diagnose human *S. japonicum* infection by detecting faecal samples (Sun et al., 2016). Xing et al. set up the real-time RPA method targeting SjR2 of *S. japonicum* to diagnose human *S. japonicum* infection by detecting faecal samples (Xing et al., 2017). Frimpong et al. used a real-time RPA method optimized targeting Dra 1 of *S. haematobium* to diagnose human *S. haematobium* infection by detecting urine samples (Frimpong et al., 2021). In the present study, we developed an LFD-RPA assay targeting SjCHGCS20 of *S. japonicum* for detecting a specific DNA (cell-free DNA) sequence in plasma of mice and goats, and the diagnostic effectiveness was compared with that of real-time PCR.

## Materials and Methods

### Plasma Sample Collection

Mice (BALB/c, 6–8 weeks old) were percutaneously infected with 40 cercariae for 15 min after counting the cercariae under a microscope. Plasma samples (0.5 ml) were collected in EDTA-K2 vacuum blood collection tubes, and the supernatant was separated after centrifugation at 3,000× rpm for 10 min at 25°C. The goat was restrained and its abdominal wool shaved. Then, goats were percutaneously infected with 300 cercariae for 20 min after counting the cercariae under a microscope. Plasma samples (2 ml) were collected in EDTA-K2 vacuum blood collection tubes from the jugular vein of each goat, and the supernatant was separated after centrifugation at 3,000× rpm for 10 min at 25°C (Guo et al., 2020). All animal experiments were conducted following the guidelines of the Committee for Care and Use of Laboratory Animals of Shanghai Veterinary Research Institute, Chinese Academy of Agricultural Sciences. The protocol was approved by the Ethics and Animal Welfare Committee of the Shanghai Veterinary Research Institute, Chinese Academy of Agricultural Sciences.

Thirty-six positive plasma samples (7 weeks post-infection) were collected from artificially infected BALB/c mice, and 48 positive plasma samples (8 weeks post-infection) were collected from artificially infected goats. Another 36 and 53 plasma samples were collected from uninfected BALB/c mice (0.5 ml) and goats (2 ml), respectively. All plasma samples were stored at -20°C.

### DNA Extraction

DNA was extracted from plasma samples of BALB/c mice (0.1 ml) and goats (0.6 ml) using a Magnetic Serum/Plasma Circulating DNA Maxi Kit (Tiangen Biotech, Beijing, China) according to the manufacturer’s protocols. The concentrations of extracted DNA were usually about 10–20 ng/μl. All extracted DNA samples were stored at -20°C. Genomic DNA samples of BALB/c mice, goats, *Haemonchus contortus*, *Toxoplasma gondii*, *Trichinella spiralis*, *Sarcocystis* sp., *Babesia*, *Fasciola gigantica*, *Paramphistomum*, and *Spirometra* were provided by the Key Laboratory of Animal Parasitology of Ministry of Agriculture. Genomic DNA samples of BALB/c mice and goats were extracted using a TIANamp Genomic DNA Kit (Tiangen Biotech) according to the manufacturer’s protocols.

### RPA Primers and Probe Design

**Figure 1:**
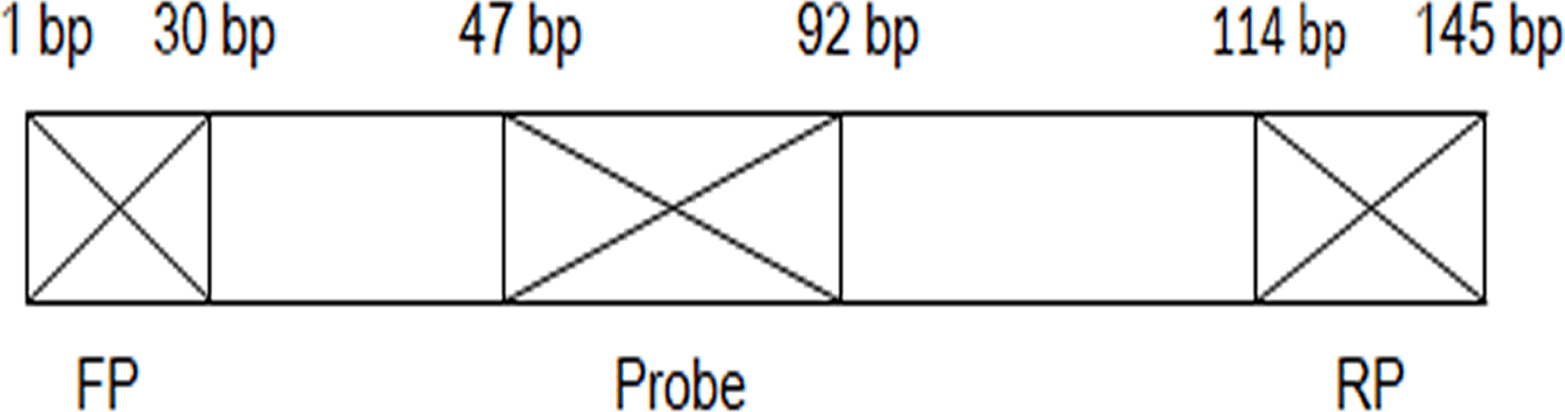
Position relationship diagram of primers and probe on the DNA sequence. FP, forward primer; RP, reverse primer; bp, base pair.

A specific *S. japonicum* DNA fragment of SjCHGCS20 was selected as the detection target sequence (GenBank: FN356222.1). The primers and probe for LFD-RPA were designed according to the TwistDX guidelines (http://www.twistdx.co.uk) and are listed in **Table 1**. A position relationship diagram of primers and probe on the DNA sequence is listed in **Figure 1**. The reverse primer was labelled with biotin at its 5′-end. The probe consisted of an oligonucleotide homologous to the *S. japonicum* target sequence, which was labelled with FAM at its 5′-end, a C3 spacer at its 3′-end, and an internal abasic nucleotide analogue (THF) with 30 bp between the 5′-end and THF, and 15 bp between the 3′-end and THF.

**Table 1:** Sequences of primers and probe used in the LFD-RPA assay.

Assay	Name	Sequence 5′–3′
	Forward primer	CCGAACACTTCAAGGAACAGTTTAGCTGGC
LFD-RPA	Reverse primer	Biotin-CTTCGTCGTTTCAGGTTAGATATAGCTTTTTG
	Probe	FAM-ATTACCCAGCTTTTCCACTCAAGCTAAATG
		/THF/AACATTGAAGTAGGC-(C3-spacer)

Biotin, antigenic label; FAM, carboxyfluorescein group; THF, an internal abasic nucleotide analogue; C3-spacer, a polymerase extension blocking group.

### LFD-RPA Assay

The total reaction volume per reaction was 50 μl, and reactions were performed using a TwistAmp nfo Kit (TwistDX Ltd, London, UK). For each reaction, 2.1 μl (10 μM) of forward primer and reverse primer, 0.6 μl (10 μM) of probe, 29.5 μl of primer-free rehydration buffer, 12.2 μl of nuclease-free water, and 1 μl template were added to the reaction pellet using the TwistAmp nfo Kit. Finally, 2.5 μl (280 mM) of magnesium acetate (MgOAc) was added to the lid of tube and mixed to start the reaction. Reactions of LFD-RPA assay were conducted for 15 min at 39°C in a dry incubator. After amplification, 5 μl of amplification product was diluted to 100 μl with the provided running buffer (Milenia Biotec, Giessen, Germany). Then, lateral flow strips (Milenia Biotec) were vertically inserted into 1.5-ml centrifuge tubes containing 100 μl of different dilutions. The results were observed after 5 min at room temperature.

In LFD-RPA assays, samples were judged positive when they simultaneously displayed a control band and a test band. Samples were judged negative when only a control band was displayed. Samples were judged invalid when only a test band or no band was displayed. A positive control [a plasmid containing the target sequence (Guo et al., 2020)] and a negative control (DNA extracted from a negative sample of a mouse or goat) were included in each run reaction.

### Optimisation of the Amount of Betaine Solution Addition

Betaine solution was added to the reaction system with an aim to reduce the secondary structure and solve the false-positive results of LFD-RPA assays (Poulton and Webster, 2018). Assays were conducted using 10 DNA respectively extracted from negative mouse and goat samples, and different amounts of betaine solution (0, 2, 5, and 8 μl) were added, respectively. Two, 5, and 8 μl of betaine solution (5 M; Sigma-Aldrich, Saint Louis, USA) were substituted for the same amount of nuclease-free water in the reaction system of RPA assays, and total volumes at 50 μl were maintained. Then, the lowest amount of betaine solution added into the reaction system was determined to effectively reduce false-positive results.

### Optimisation of the LFD-RPA System

The amplification temperature, reaction duration, and product dilution are three important factors in the LFD-RPA assay. The LFD-RPA assays were conducted using plasmid (2.6 fg) containing the target sequence as template to optimize the amplification conditions. The amplification temperatures were tested at 25°C, 30°C, 35°C, 37°C, 39°C, 42°C, and 45°C, respectively. The amplification durations were tested at 5, 10, 15, and 20 min at 39°C, respectively. Amplification products were diluted 10-, 20-, 50-, and 100-fold with running buffer and tested at 39°C for 15 min to determine the optimal dilution.

### Analysis of the Lower Limit of Detection and Cross-Reactivity of LFD-RPA and Real-Time PCR Assays

The total reaction system volume of real-time PCR assays was 20 μl. It included 10 μl of 2×ChamQ Universal SYBR qPCR Master Mix (Vazyme Biotech, Nanjing, China), 0.4 μl (10 μmol/l) of each primer, 5.2 μl ddH_2_O, and 4 μl (~20 ng) DNA template. The reaction procedure comprised an initial denaturing step at 94°C for 30 s, followed by 40 cycles at 94°C for 15s, 58°C for 34s, and 72°C for 10 s, and a melting curve step, comprising denaturation at 95°C for 15 s, annealing at 60°C for 15 s, and extension at 72°C for 15 s using a qTOWER 3G instrument (Analytik Jena AG, Jena, Germany) (Guo et al., 2020). Besides, the LFD-RPA and real-time PCR assays detect the same DNA fragment of *S. japonicum*.

The positive plasmid containing the target sequence was diluted to 2.6 ng/μl, and a 10-fold gradient dilution series to 0.26 fg/μl was used to establish the lower limit of detection of the LFD-RPA and real-time PCR assays. The cross-reactivity of the LFD-RPA assay with other parasites was evaluated using genomic DNA (200–300 ng) from eight additional parasites (*Haemonchus contortus*, *Toxoplasma gondii*, *Trichinella spiralis*, *Sarcocystis* sp., *Babesia*, *Fasciola gigantica*, *Paramphistomum*, and *Spirometra*).

### Detection of Schistosomiasis Japonica in Mice by LFD-RPA and Real-Time PCR Assays

The sensitivity of LFD-RPA and real-time PCR assays was calculated using DNA templates extracted from 36 infection-positive plasma samples from mice. The specificity of LFD-RPA and real-time PCR assays was calculated by detecting DNA templates extracted from 36 mice without schistosome infection.

### Detection of Schistosomiasis Japonica in Goats by LFD-RPA and Real-Time PCR Assays

A total of 48 positive plasma samples and 53 negative plasma samples from goats were analysed by the two methods. The sensitivity of LFD-RPA and real-time PCR assays was calculated by detecting DNA templates from positive plasma samples. The specificity of LFD-RPA and real-time PCR assays was calculated by detecting DNA templates from goats without schistosome infection.

### Data Analysis

Stata/SE 12.0 was used to calculate the 95% confidence interval (CI) for both sensitivity and specificity. Analysis of the LFD-RPA and real-time PCR assay results was conducted by kappa and chi-square tests using SPSS Statistics (Version 20). The coefficient of *k* values was scored as follows: negligible (0–0.20), weak (0.21–0.40), moderate (0.41–0.60), substantial (0.61–0.80), and excellent (0.81–1.00). A *p*-value <0.05 was considered statistically significant.

## Results

### Establishment and Optimisation of the LFD-RPA Assay

The results showed that the false-positive rate was 20% (2/10) without adding betaine solution. The false-positive rate was 10% (1/10) after adding 2 μl betaine solution. No false positive was appeared after respectively adding 5 and 8 μl betaine solution in detecting mouse negative samples. The false-positive rates were respective 30% (3/10), 20% (2/10), 0 (0/10), and 0 (0/10) after adding 0, 2, 5, and 8 μl betaine solution in detecting goat negative samples. Thus, 5 μl was set as the optimum addition amount of betaine solution in the subsequent detection reactions.

**Figure 2:**
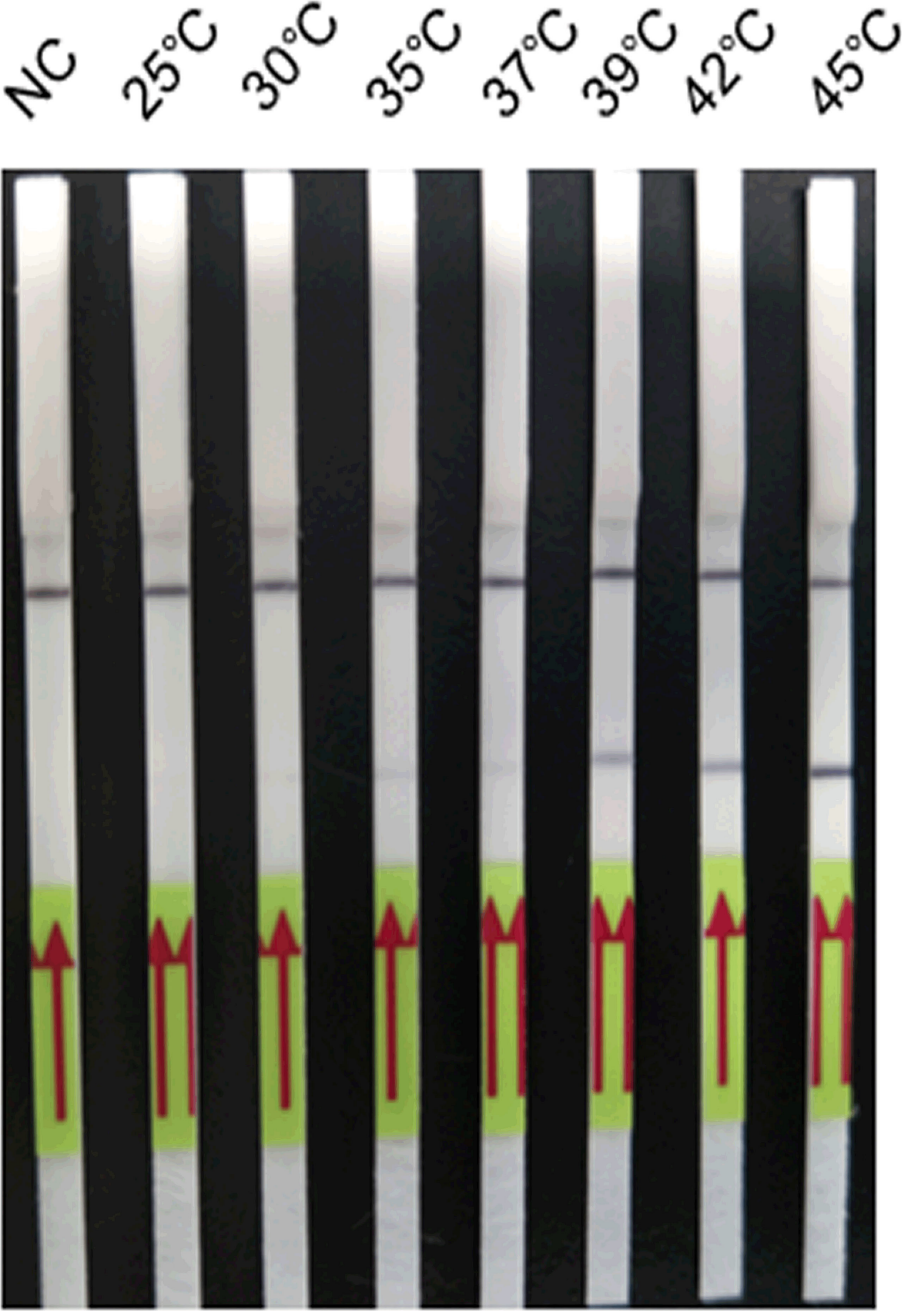
Optimisation of the amplification temperature of the LFD-RPA assay. The LFD-RPA assay results are positive over a wide range of temperatures from 35°C to 45°C. The samples of 25°C–45°C were all 2.6 fg/μl positive plasmid. Negative control (NC), DNA extracted from negative samples and amplification at 45°C. The red arrows stand for the direction of reaction solution diffusing in the lateral flow dipstick.

Analysis of amplification temperature showed that the reaction could be conducted over a wide range of temperatures from 35°C to 45°C (**Figure 2**). The test bands gradually became bright when the amplification temperature was increased, and a clearly visible test band could be observed from 39°C to 45°C. Thus, 39°C was set as the optimum amplification temperature.

**Figure 3:**
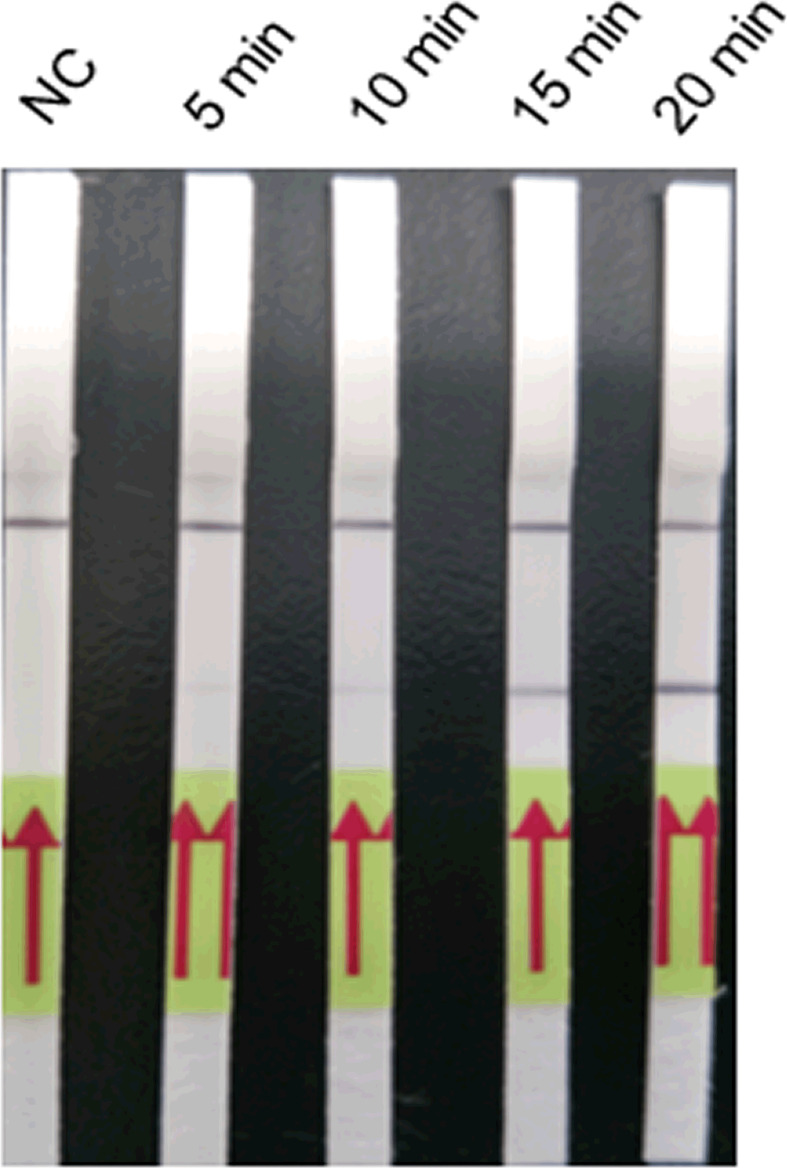
Optimisation of the amplification duration of the LFD-PCR assay. The LFD-RPA assay results are positive for 5, 10, 15, and 20 min. The samples of 5, 10, 15, and 20 min were all 2.6 fg/μl positive plasmid. Negative control (NC), DNA extracted from negative samples and amplification duration for 20 min. The red arrows stand for the direction of reaction solution diffusing in the lateral flow dipstick.

The LFD-RPA assay could be conducted over 5, 10, 15, and 20 min at 39°C (**Figure 3**). The bands were weak after 5 and 10 min but were clearly visible at 15 and 20 min. Thus, 15 min was determined as the optimum amplification duration.

**Figure 4:**
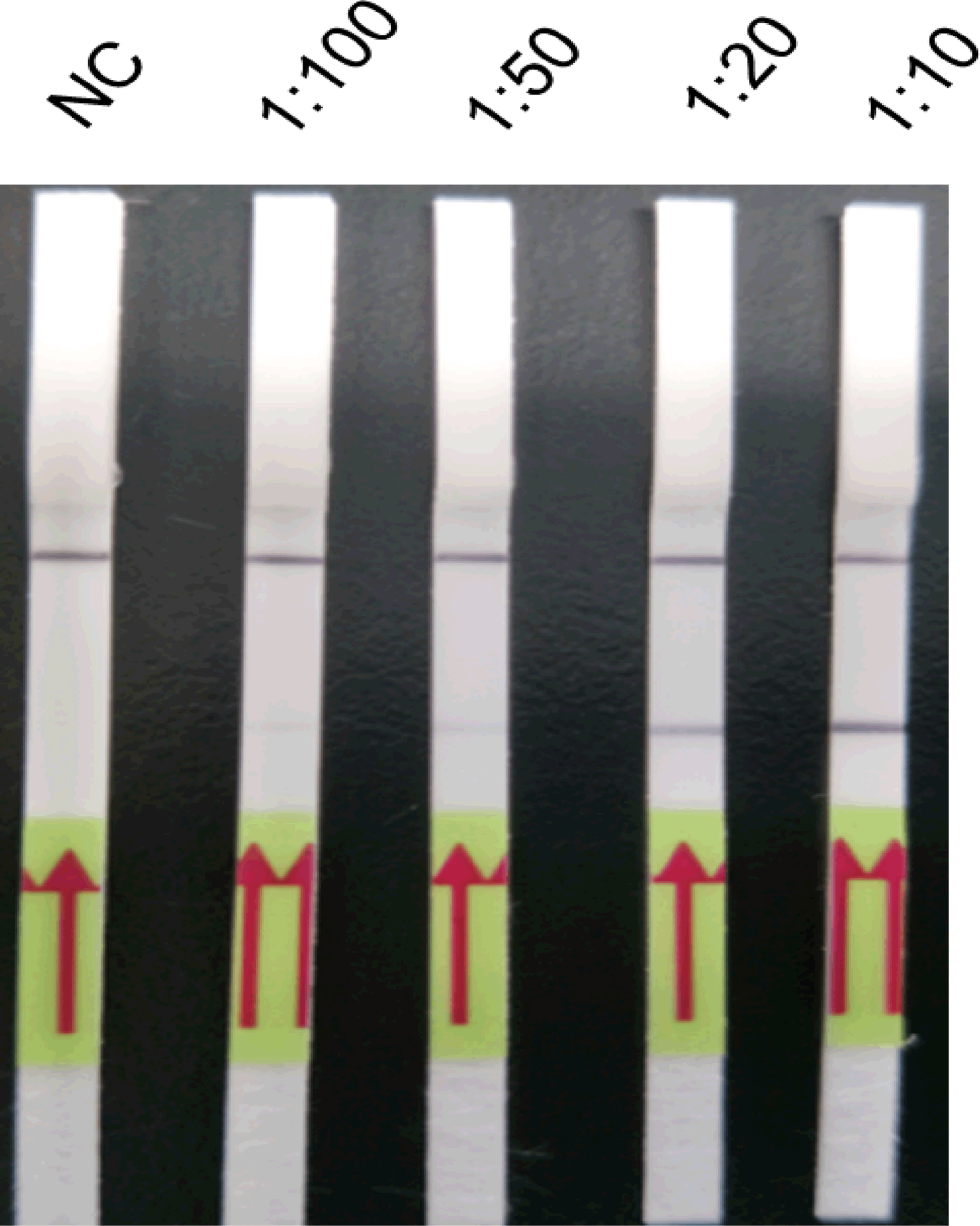
Optimisation of the amplification product dilution of the LFD-PCR assay. The LFD-RPA assay results are positive at amplification product dilutions of 1:10, 1:20, 1:50, and 1:100. A clearly visible test band can be observed at 1:10 and 1:20 dilutions, but only a weak test band is visible at 1:50 and 1:100 dilutions. The samples of amplification product dilutions of 1:10, 1:20, 1:50, and 1:100 were all 2.6 fg/μl positive plasmid. Negative control (NC), DNA extracted from negative samples and amplification product in a dilution of 1:10. The red arrows stand for the direction of reaction solution diffusing in the lateral flow dipstick.

Amplification products were diluted by 1:10, 1:20, 1:50, and 1:100 with running buffer (**Figure 4**). Test bands were observed at all four dilutions, and a clearly visible test band was observed at 1:20 and 1:10 dilutions. By contrast, only weak test bands were observed at 1:50 and 1:100 dilutions. Thus, 1:20 was determined as the optimum dilution.

### Comparison of the Lower Limit of Detection and Cross-Reactivity of LFD-RPA and Real-Time PCR Assays

**Figure 5:**
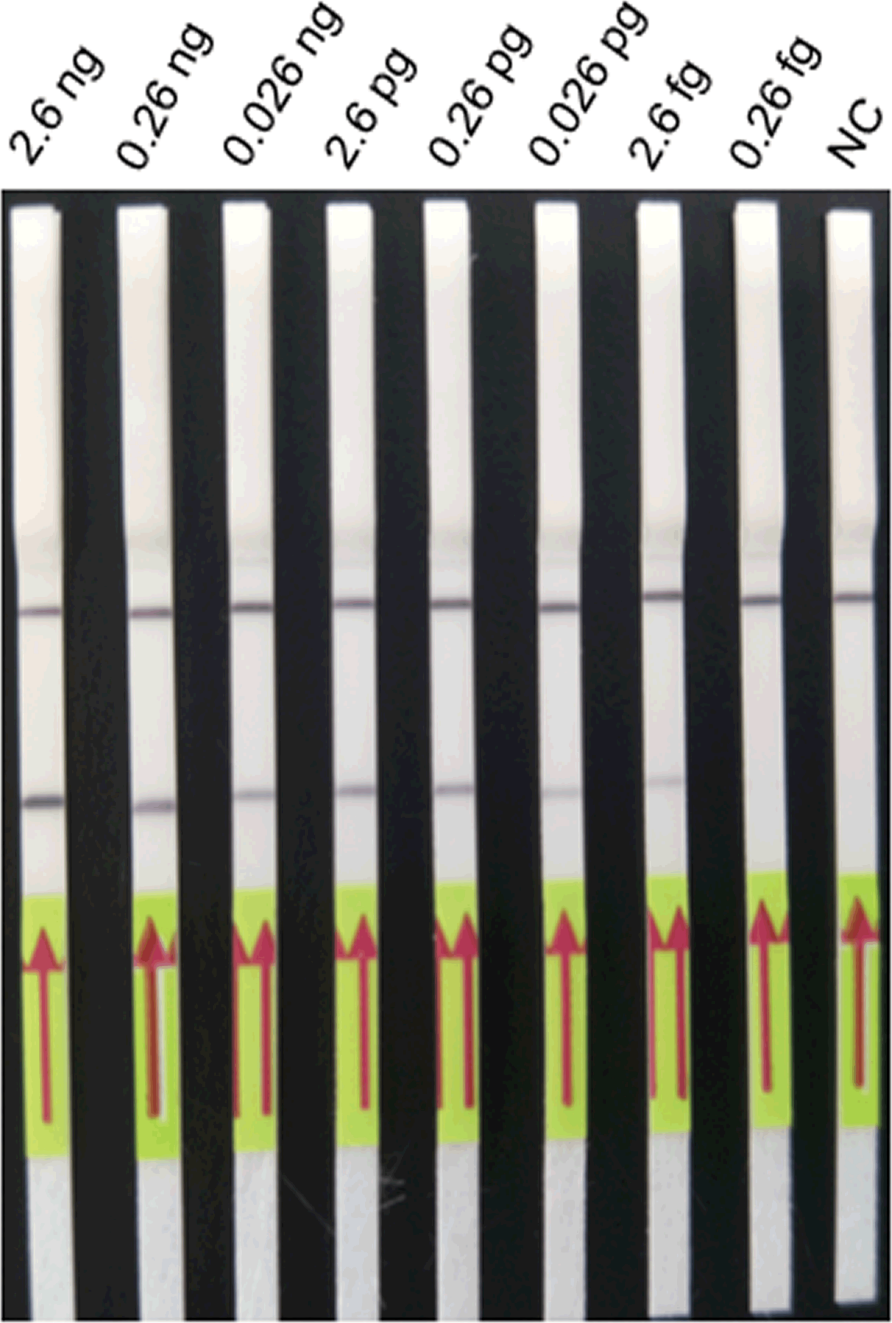
The lower limit of detection of the LFD-RPA assay. Positive plasmid containing the *S. japonicum* target sequence at concentrations from 2.6 nanogram (ng)/μl to 0.26 fg/μl was used to determine the minimum detection concentration in the LFD-RPA assay. The minimum detection concentration is 2.6 fg/μl. Negative control (NC), DNA extracted from negative samples. The red arrows stand for the direction of reaction solution diffusing in the lateral flow dipstick.

**Figure 6:**
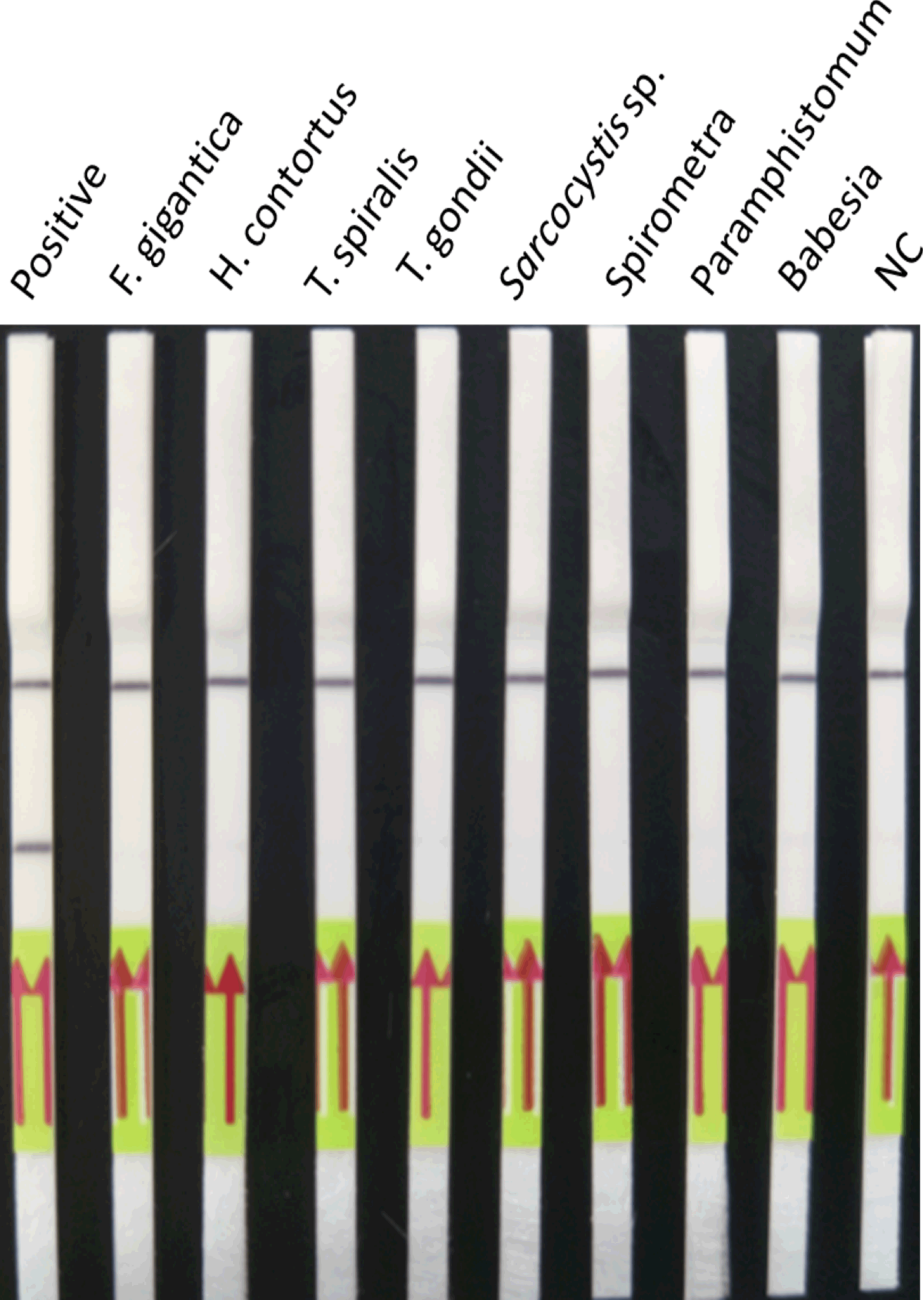
Cross-reactivity of the LFD-RPA assay. The LFD-RPA assay detects *S. japonicum* exclusively and exhibits no cross-reactivity with genomic DNA from eight other parasites. Negative control (NC), DNA extracted from negative samples. The red arrows stand for the direction of reaction solution diffusing in the lateral flow dipstick.

The results showed that the lower limit of detection of the LFD-RPA assay was 2.6 fg plasmid DNA containing the target DNA of *S. japonicum* (~39 fg genomic DNA of *S. japonicum*) (**Figure 5**) compared with 0.26 fg of *S. japonicum* target DNA (~3.9 fg genomic DNA of *S. japonicum*) for the real-time PCR assay. No amplification was observed using genomic DNA of these eight kinds of other parasites and genomic DNA of the mouse and goat as templates by the two methods (**Figure 6**).

### Sensitivity and Specificity of LFD-RPA and Real-Time PCR Assays for Schistosomiasis Japonica Detection in Mice

The results of LFD-RPA and real-time PCR showed that there was no gene amplification from the genomic DNA of mice, and they could be used to detect *S. japonicum* infection in mice. The results showed that the sensitivity was 97.22% (35/36, 95% CI, 85.47%–99.93%) and the specificity was 100% (36/36, 95% CI, 90.26%–100%). The detection results for real-time PCR showed that the sensitivity and specificity were both 100% (36/36, 95% CI, 90.26%–100%). The chi-square test results revealed that there was no significant difference (*p* = 0.314) in sensitivity between the LFD-RPA and real-time PCR assays for detecting schistosomiasis japonica in mice, and the specificity was 100% for both methods.

### Sensitivity and Specificity of LFD-RPA and Real-Time PCR Assays for Schistosomiasis Japonica Detection in Goats

The results of LFD-RPA and real-time PCR showed that there was no gene amplification from genomic DNA of goats, and they could be used to detect *S. japonicum* infection in goats. The results showed that the sensitivity was 93.75% (45/48, 95% CI, 82.80%–98.69%) and the specificity was 100% (53/53, 95% CI, 93.28%–100%). The sensitivity and specificity of the real-time PCR assay was 97.92% (47/48, 95% CI, 88.93%–99.95%) and 100% (53/53, 95% CI, 93.28%–100%), respectively, in goats. The kappa test results showed that the LFD-RPA assay was similar (*k* = 0.484) with the real-time PCR assay in terms of sensitivity. The chi-square test results revealed no significant difference (*p* = 0.307) in sensitivity between the LFD-RPA and real-time PCR assays for detecting schistosomiasis japonica in goats, and the specificity was 100% for both methods.

## Discussion

In recent decades, the incidence of schistosomiasis japonica has decreased significantly in China following extensive efforts to prevent and control this disease. However, there are still some risk factors in low-prevalence and low-intensity infection areas in China, resulting in disease transmission. People and animals in those areas can still be at the risk of infection (Zhou et al., 2005b; Weerakoon et al., 2018; Okeke et al., 2020). Assay sensitivity and specificity are crucial for accurate diagnosis of schistosomiasis japonica; lower sensitivity and specificity may result in disease transmission due to missed diagnosis (Magalhaes et al., 2020).

Recombinase polymerase amplification has a wide range of applications, and it is especially suitable for detection in the field or in low-resource settings (Xing et al., 2017; Poulton and Webster, 2018; Li et al., 2019; Frimpong et al., 2021). The main advantage of RPA is that it can achieve isothermal amplification; hence, high temperatures are not needed. The RPA responds quickly, and results can be generated within 20 min. The main reagents for RPA are provided in a lyophilised form and have high stability for at least 12 months at room temperature; hence, they do not require a cold storage system during short-term transportation. In addition, analysis of RPA results is flexible and diverse, and LFD-RPA is very practical for diagnosing diseases (Molina-Gonzalez et al., 2020; Zhai et al., 2020).

In previous studies, human schistosomiasis japonica was detected by LFD-RPA assay. The lower limit of detection was 5 fg of *S. japonicum* DNA, and the sensitivity and specificity were 92.86% (13/14) and 100% (31/31), respectively (Sun et al., 2016). Xing et al. established a real-time RPA method for detecting human schistosomiasis japonica, and the lower limit of detection was 0.9 fg *S. japonicum* DNA. The sensitivity and specificity were both 100% (30/30; 30/30) (Xing et al., 2017). The detection templates were genomic DNA of eggs from human stool samples in the above two methods. However, domestic animals were usually fed in a large group and collecting faeces of each domestic animal was a difficult job. It was also easy to cause stool sample contamination during the process. Thus, it was not suitable to use stool samples for detecting although it was non-invasive. Xu et al. used a LAMP method for detecting human schistosomiasis japonica, and the detection templates were *S. japonicum*—specific DNA in serum samples collected from human. The lower limit of detection was 0.08 fg *S. japonicum* DNA, and the sensitivity and specificity were 96.7% (29/30) and 100% (20/20), respectively (Xu et al., 2010). In this study, the detection templates were cell-free DNA from plasma samples of mice and goats.

There was no previous report for the diagnosis of domestic animal schistosomiasis by the RPA method. At present, only nested-PCR and real-time PCR methods were reported for diagnosing schistosomiasis japonica in domestic animals. The nested-PCR method was used to detect the target DNA of *S. japonicum* from buffaloes and goats at day 3 post-infection. The sensitivity in buffaloes was 92.30% (24/24), and the specificity was 97.60% (41/42). However, it needed two rounds of PCR, which takes a long time (3 h) and increases the risk of contamination (Zhang et al., 2017). The sensitivity and specificity of real-time PCR were 98.74% (157/159, 95% CI: 95.53%–99.85%) and 100% (94/94, 95% CI: 96.15%–100%) in goats, respectively (Guo et al., 2020). It was also time-consuming (1.5 h) and requires the use of a real-time fluorescent quantitative PCR instrument, which was hindered for diagnosing animal schistosomiasis japonica in resource-limited settings. This study was the first to diagnose schistosomiasis japonica in domestic animals by LFD-RPA assay and reduce the reaction time significantly (15 min), providing an alternative means for diagnosing animal schistosomiasis japonica in low-resource settings.

In the present study, an RPA assay for schistosomiasis japonica was successfully set up in which amplification of a specific DNA fragment was achieved, and the results could be easily detected by LFD. The RPA assay could be completed at 39°C in 15 min. The amplification product was diluted 20-fold with running buffer to a total volume of 100 μl and incubated for 5 min at room temperature, and the results were observed by LFD. The lower limit of detection of the LFD-RPA assay was 2.6 fg of *S. japonicum* target DNA (~39 fg genomic DNA of *S. japonicum*), higher than that of the real-time PCR (~3.9 fg genomic DNA of *S. japonicum*). Although the LFD-RPA assay is not as sensitive as real-time PCR at present, it could be improved through further optimisation, by modifying RPA probes, primers, target sequences, etc.

Like PCR methods, the LFD-RPA assay can also form primer dimers, probe-primer dimers, and hairpin structures, thereby generating false positives. Betaine solution is often used in nucleic acid amplification to prevent the formation of secondary structures such as hairpins, especially for DNA with a high GC content (Kang et al., 2005; Poulton and Webster, 2018). In the present study, betaine solution (Sigma-Aldrich) was added to the LFD-RPA reaction system to prevent the formation of secondary structures and improve reaction accuracy (Poulton and Webster, 2018). Specifically, 5 μl of nuclease-free water was replaced with the same amount of betaine solution (5 M) to a total volume of 50 μl, to reduce non-primary structures and minimise false positives.

The sensitivity and specificity of the LFD-RPA assay for schistosomiasis japonica detection were evaluated in a typical laboratory animal (mouse). The results showed that the sensitivity was 97.22% (35/36, 95% CI, 85.47%–99.93%) and the specificity was 100% (36/36, 95% CI, 90.26%–100%). There was no significant difference (*p* = 0.314) in sensitivity between the LFD-RPA and real-time PCR assays; the specificity was 100% for both. The LFD-RPA assay was then used to detect schistosomiasis japonica in a typical domestic animal (goat). The results showed that the sensitivity was 93.75% (45/48, 95% CI, 82.80%–98.69%) and the specificity was 100% (53/53, 95% CI, 93.28%–100%). The LFD-RPA assay was in reasonable agreement (*k* = 0.484) with the real-time PCR assay, and there were no significant differences (*p* = 0.307) in sensitivity or specificity for detecting schistosomiasis japonica in goats. In addition, the LFD-RPA assay exhibited no cross-reactivity with genomic DNA from eight different parasites.

In summary, the LFD-RPA assay is an alternative method for the diagnosis of animal schistosomiasis japonica in low-resource settings. In previous studies, the RPA presented better consistency with the stool-based tests than IHA and ELISA (Sun et al., 2016; Xing et al., 2017). Meanwhile, it possesses high sensitivity and specificity, simplicity in operation, quick display of results, and no requirements for advanced instrumentation compared with some traditional diagnosis methods. It could be operated by local veterinarians or related technicians who are less knowledgeable on molecular diagnosis. For example, with the application of the LFD-RPA method for rapid viral detection of COVID-19, prospective applicability in resource-limited and decentralized laboratories will become possible (Shelite et al., 2021). However, this method also shows some shortcomings. The nucleic acid extraction process is relatively cumbersome. The lid needs to be opened for product LFD detection after the amplification is completed, and this will increase aerosol contamination in the environment. In addition, the kit is relatively expensive. Therefore, further work is needed to simplify the nucleic acid extraction process, use a fully enclosed device to avoid aerosol contamination, and improve its practicality in low-resource settings.

## Conclusions

An LFD-RPA assay was developed for schistosomiasis japonica detection in mice and goats, which has advantages over existing detection methods. It benefits from high sensitivity and specificity and exhibits no cross-reactivity with eight other parasites. It provides an alternative method for the diagnosis of animal schistosomiasis japonica in low-resource settings.

## Data Availability Statement

The datasets presented in this study can be found in online repositories. The names of the repository/repositories and accession number(s) can be found in the article/supplementary material.

## Ethics Statement

The animal study was reviewed and approved by the Animal Care and Use Committee at the Shanghai Veterinary Research Institute, Chinese Academy of Agricultural Sciences.

## Author Contributions

QG, CC, KRZ, ZS, and YY performed the experiments. QG and YH drafted the manuscript. YH, ZF, and JJL designed the study and critically revised the manuscript. QG, JML, and YY performed the statistical analysis. CX, WT, XC, XS, and KKZ provided most of the materials needed. All authors contributed to the article and approved the submitted version.

## Funding

This study was supported by the Natural Science Foundation of Shanghai (19ZR1468900), the Shanghai Science and Technology Promotion Agriculture Innovation Program (2019 No. 3-3), the National Key Research and Development Program of China (2017YFD0501306), the National Natural Science Foundation of China (31402192, 31872256), and the Scientific and Technical Innovation Project of the Chinese Academy of Agricultural Sciences.

## Conflict of Interest

The authors declare that the research was conducted in the absence of any commercial or financial relationships that could be construed as a potential conflict of interest.

## Publisher’s Note

All claims expressed in this article are solely those of the authors and do not necessarily represent those of their affiliated organizations, or those of the publisher, the editors and the reviewers. Any product that may be evaluated in this article, or claim that may be made by its manufacturer, is not guaranteed or endorsed by the publisher.

## Abbreviations

ELISA, enzyme-linked immunosorbent assay; MHT, miracidium hatching test; IHA, indirect hemagglutination assay; LAMP, loop-mediated isothermal amplification; RPA, recombinant enzyme polymerase amplification; LFD, lateral flow dipstick; LFD-RPA, lateral-flow stripe recombinase polymerase amplification.
